# Foamy virus for efficient gene transfer in regeneration studies

**DOI:** 10.1186/1471-213X-13-17

**Published:** 2013-05-03

**Authors:** Shahryar Khattak, Tatiana Sandoval-Guzmán, Nicole Stanke, Stephanie Protze, Elly M Tanaka, Dirk Lindemann

**Affiliations:** 1 Max Planck Institute of Molecular Cell Biology and Genetics, Pfotenhauerstr 108, 01307 Dresden, Germany; 2 DFG-Center for RegenerativeTherapies Dresden, Technische Universität Dresden, Fetscherstr. 105, 01307 Dresden, Germany; 3 Institute of Virology, Medical Faculty, Technische Universität Dresden, Fetscherstr 74, 01307 Dresden, Germany

**Keywords:** Foamy virus, Regeneration, Salamander, in vivo gene transfer

## Abstract

**Background:**

Molecular studies of appendage regeneration have been hindered by the lack of a stable and efficient means of transferring exogenous genes. We therefore sought an efficient integrating virus system that could be used to study limb and tail regeneration in salamanders.

**Results:**

We show that replication-deficient foamy virus (FV) vectors efficiently transduce cells in two different regeneration models in cell culture and in vivo. Injection of EGFP-expressing FV but not lentivirus vector particles into regenerating limbs and tail resulted in widespread expression that persisted throughout regeneration and reamputation pointing to the utility of FV for analyzing adult phenotypes in non-mammalian models. Furthermore, tissue specific transgene expression is achieved using FV vectors during limb regeneration.

**Conclusions:**

FV vectors are efficient mean of transferring genes into axolotl limb/tail and infection persists throughout regeneration and reamputation. This is a nontoxic method of delivering genes into axolotls in vivo/ in vitro and can potentially be applied to other salamander species.

## Background

Limb regeneration of salamanders has fascinated scientists for several decades. Salamanders, Newts and Axolotls, are used as model animals in limb regeneration studies. Regeneration of missing structures is achieved by blastema, a pool of restricted progenitors that is formed after amputation [1].

Electroporation of DNA is the fastest and efficient method to introduce exogenous transgenes into salamander limb and spinal cord in vivo but this expression is lost during regeneration as the electroporated DNA is episomal [2].

Infection of cells both in cell culture and in vivo by modified viruses has been a powerful means of expressing exogenous genes in various experimental systems. For example, retroviral infection has been crucial for the molecular analysis of chicken limb development [3]. A corresponding molecular analysis of limb regeneration in salamanders has been limited due to a paucity in gene transduction methods. Vaccinia and adenovirus have been used in limb regeneration studies, but their toxicity and non-integration phenotype limits their effectiveness [4,5]. Similarly, pseudotyped retroviruses have been used in cultured cells [5-7] and a recent report showing their use in vivo but the issue of viral silencing after second round of regeneration was not investigated [8]. We therefore sought a virus system that efficiently and stably infects salamander cells in vitro and in vivo and does not require pseudotyping and is not prone to silencing during initial and second round of regeneration.

Foamy viruses (FV) are a special type of retroviruses that are endemic to most non-human primates, horses, cattle and cats [9]. They were only lately successfully introduced into the repertoire of vector systems for the correction of inherited diseases, in particular of the hematopoietic lineage, in the mammalian system [10,11]. However, they have proven to be an efficient, non-toxic and stably integrating gene delivery vector system for mammalian cells. Some of their unique features, including their apparent apathogenicity, infectious particle-associated DNA genome, extremely broad host range as well as their efficient transduction capability for hematopoietic stem cells (HSC), make them one of the most promising tools for various gene therapeutic approaches [11].

One hallmark of FVs is their extremely broad tropism, including even very distantly related organisms as reptiles or birds [12,13]. The nature of the broadly expressed, and potentially evolutionary conserved, cellular receptor(s) of FV glycoprotein-mediated attachment and entry remains poorly characterized. Though, several lines of evidence from recent publications suggest that proteoglycans and heparin sulfate function particularly as important attachment factors for FV Env-mediated host cell infection, additional uncharacterized molecules appear to be essential for fusion of viral and cellular lipid membranes during uptake of virions [14,15]. Based on this feature we tested whether salamander cells would be transduced by FV vectors in vitro and in vivo. We also compared their transduction profile to that of lentiviral (LV) vectors pseudotyped with the vesicular stomatitis virus glycoprotein G (VSV-G), which has gained favor in gene delivery methods [16].

## Results and discussion

**Figure 1:**
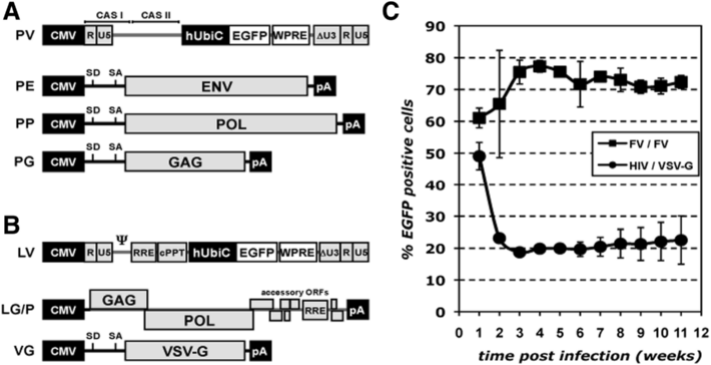
**Foamy virus displays high efficiency of transduction with stable expression in cultured newt cells. A**+**B**) Schematic outline of the FV (**A**) and lentiviral (**B**) vector systems used. FV or LV components are indicated in grey, heterologous transcriptional control elements in black, and the transgene and posttranslational control element in white. **C**) The Newt myogenic cell line A1, was transduced with FV (FV/FV, solid squares) or LV (HIV/VSV-G, solid circles) vector particles expressing a hUbiC promoter-driven EGFP transgene at a MOI of 130. In weekly intervals the percentage of EGFP expressing cells was determined by flow cytometry. The initial rate of expression was similar for both viruses but FV transduced cells increased over time, plateauing at 3 weeks post-infection at approximately 75% expressing cells. In contrast, a large number of LV transduced cells lost expression after initial infection and plateaued at 20% expressing cells. PFV based transfer vector (PV) and packaging constructs for PFV glycoprotein (PE, ENV), PFV polymerase (PP, POL) and PFV capsid (PG, GAG); HIV-1 based transfer vector (LV) and packaging constructs for HIV-1 capsid (GAG) and polymerase (POL) (LG/P) and VSV glycoprotein (VG, VSV-G). CMV: cytomegalo virus immediate early enhancer – promoter; R: long terminal repeat (LTR) repeat region; U5: LTR unique 5’ region; ΔU3: enhancer-promoter deleted LTR unique 3’ region; CASI+II: FV cis-acting sequences I and II; hUbiC: human ubiqutin C promoter; WPRE: Woodchuck hepatitis virus posttranscriptional regulatory element; : HIV-1 packaging sequence; RRE: rev-responsive-element; cPPT: central poly purine tract; SD: splice donor; SA: splice acceptor.

To test and compare transduction efficiencies of Foamy virus (FV) and Lentivirus (LV) vectors in salamanders, FV and LV vector particles were generated by transient transfection of 293T cells with transfer vectors (PV, LV) containing an identical human ubiquitin C (UBIC) promoter driven EGFP marker gene cassette including a WPRE posttranscriptional element, and the corresponding packaging expression constructs (PE, PP, PG; LG/P, VG) (Figure 1A, B). We first tested the infectability of cultured cell lines derived from the newt and the axolotl limb [5,17]. Target cells were exposed to vector particles at a titer of 1.74x10^6^ IU/ml (FV and LV) for 8 hours with an MOI (multiplicity of infection) of 130 and analyzed in weekly intervals by FACS analysis for the percentage of EGFP-positive cells (Figure 1C). In one week, the newt and axolotl cells undergo approximately two doublings. One week after viral transduction, both the LV- and FV-transduced cells showed close to 50% GFP-expressing cells. By four weeks, the FV-transduced cell preparation showed close to 75% EGFP-expressing cells and remained stably at this level over 11 weeks in culture. In contrast, the LV-transduced cells decreased stably to 20% EGFP-expressing cells. Furthermore, using higher concentration/titer of viruses or different ratios of vector particles to target cells, a similar decline in LV infectivity was observed suggesting that the decline/inefficiency of LV infection cannot be circumvented by using more infectious particles (data not shown).

**Figure 2:**
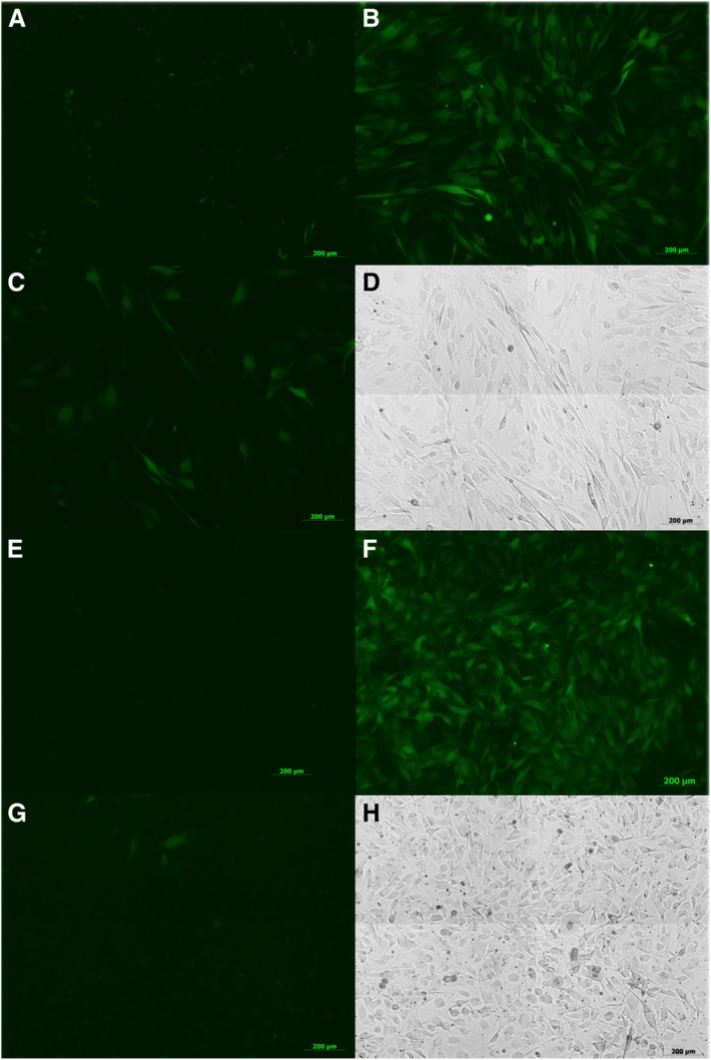
**Foamy virus vectors transduce both newt and axolotl cell cultures.** Newt A1 (**A**-**D**) and Axolotl blastema (**E**-**H**) cells were infected with FV and LV vectors encoding EGFP and epifluorescence images taken 3 weeks post-transduction. **A**. Uninfected newt cells. **B**. FV infected newt cells. **C**. LV infected newt cells. **D**. Phase contrast image of culture from C. **E**. Uninfected axolotl cells. **F**. FV infected axolotl cells. **G**. LV infected axolotl cells. **H**. Phase contrast image of culture from G. Scale Bars: 200 μm.

Figure 2 shows fluorescence images of EGFP expressing newt (Figure 2A-D) and axolotl cell cultures (Figure 2E-H) at 21 days post transduction with FV and LV vectors. The reason for the initial decline in the number of EGFP-positive cells in samples transduced with LV vectors and increase in FV vector transduced samples within the first three weeks post transduction is currently unclear. A silencing of integrated LV vector genomes and not FV vector genomes in individual cells is one possible explanation. Alternatively, it might be the result of the differential capability of LV and FV vectors to integrate their reverse transcribed genomes in interphase chromatin. The LV vector derived initial high number of EGFP expressing cells can be a consequence of reverse transcribed HIV genomes actively imported into the nucleus of cells infected during interphase and resulting in expression from episomal vector DNA genomes. The subsequent decline within the first 3 weeks after transduction may then be a consequence of inefficient viral integrase-mediated LV vector insertion into the host genome. In contrast, FV vectors are known to require mitosis for access to host chromatin and can reside in a latent state at the centrosome of non-dividing cells for weeks [18,19]. Thus, an increase of the percentage of EGFP expressing cells within the first 3 weeks after transduction might be the result of efficient integration of FV vector genomes only after individual mitotic divisions of individual cells. For the majority of target cells this might take up to three weeks.

**Figure 3:**
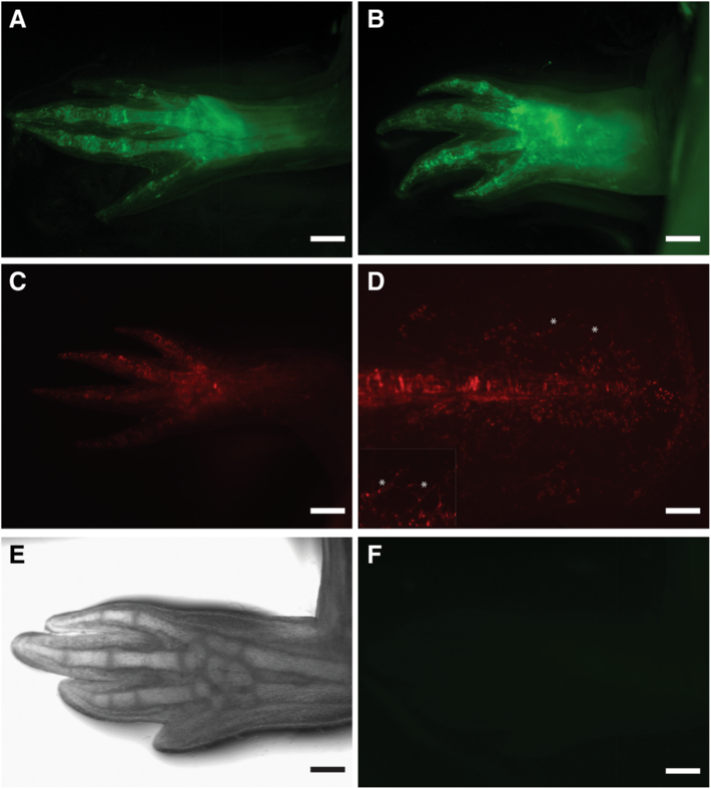
**In vivo infection of regenerating limbs with foamy versus lentivirus vector particles.** Concentrated FV or LV vector particles were injected into 5-day limb blastemas, which were then allowed to regenerate. Images were taken 28 days post-injection. **A**. FV vector particle (EGFP) injected limb. **B**. FV vector particle injected limb that had regenerated, and then was reamputated and regenerated a second time showing stability of expression. **C**. FV vector particle (DsRed) injected limb. **D**. FV vector particle (DsRed) injected tail. Asterisk point to labeled blood vessels. The inset shows high magnification of the fin where blood vessels are labeled. **E**, **F**. LV vector particle (EGFP) injected limb in bright field (**E**), and fluorescence (**F**) shows no fluorescence signal. Scale Bars: A,C: 500 μm B,D,E,F: 1 mm.

We next wanted to test the ability of the viruses to infect limb tissue in vivo. Concentrated virus preparations were prepared by centrifugation and subsequently injected in mature limbs of axolotls. We did not observe any EGFP labeling in these limbs injected with LV or FV particles (data not shown). Since FV require mitosis to access their host genome, we injected LV and FV particles into 5 day old axolotl forelimb blastemas. Injection of 0.5 to 2 μl of concentrated FV particles (titer of 1.44×10^8^ IU/ml) consistently yielded labeling of many limb cells (Figure 3A). To test the stability of transgene expression, we allowed the limbs to fully regenerate and then re-amputated the limbs. After 5 weeks of second round regeneration, we continued to observe robust expression of EGFP (Figure 3B). Similarly, tail and limb blastema were injected with FV particles containing UBIC promoter driving DsRed and allowed to regenerate (Figure 3C, D). In contrast, we observed virtually no gene expression after injection of a similar volume of the highest concentration LV preparations that we could achieve (titer of 1.02×10^8^ IU/ml) (Figure 3E, F).

**Figure 4:**
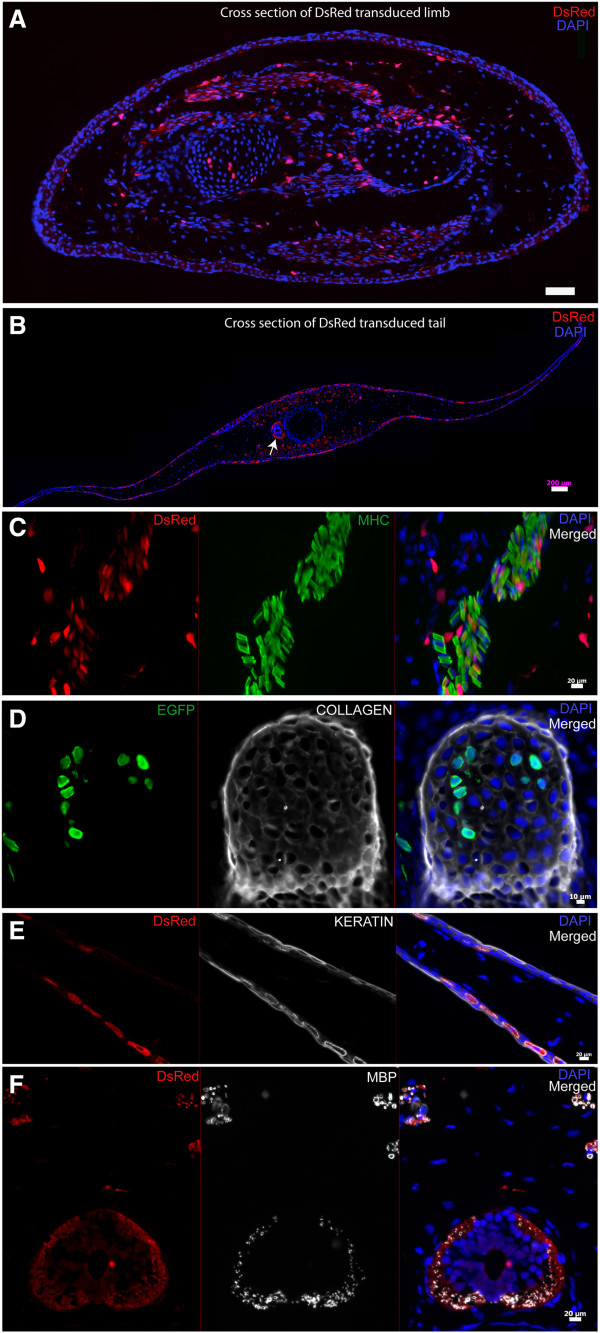
**Foamy virus vector transduce muscle, cartilage, dermis, Schwann cells in the regenerating axolotl limb and tail. A**. Cross section of limb shown in Figure 3C (transduced with FV-DsRed) showing wide spread DsRed expression. **B**. Cross section of tail shown in Figure 3D (transduced with FV-DsRed) showing wide spread DsRed expression. Arrow points to the spinal cord. **C**. Immunofluorescence staining for muscle specific myosin heavy chain (MHC) in limb (transduced with FV-DsRed) showing endogenous DsRed expression (left), MHC staining (middle) and merged of the two images is shown on the right. (DAPI=blue). **D**. Immunofluorescence staining for collagen in limb (transduced with FV-EGFP) endogenous EGFP expression is on the left, collagen staining (middle) and merged of the two images is shown on the right. (DAPI=blue). **E**. Immunofluorescence staining for keratin in tail (transduced with FV-DsRed). The image shown is a blow up of the tail fin region showing excellent colocalization of endogenous DsRed expression (left) with keratin immunostaining (middle). **F**. Immunofluorescence staining for myelin basic protein (MBP) in tail (transduced with FV-DsRed). The image depicted here shows the spinal cord and associated peripheral nerve tracts showing endogenous DsRed expression (left), MBP staining (middle) and merged of the two images on the right. Scale bars: A: 100 μm, B: 200 μm, C,E,F: 20 μm, D: 10 μm.

To determine the specific cell types that were labeled we cross sectioned the infected, regenerated limbs and performed immunostaining. We observed EGFP/DsRed expression that co-localized with immunostaining for muscle specific myosin (muscle), collagen (cartilage), keratin (dermis), myelin basic protein (Schwann cells and nerve tracts) and blood vessels (Figure 3D, Figure 4).

**Figure 5:**
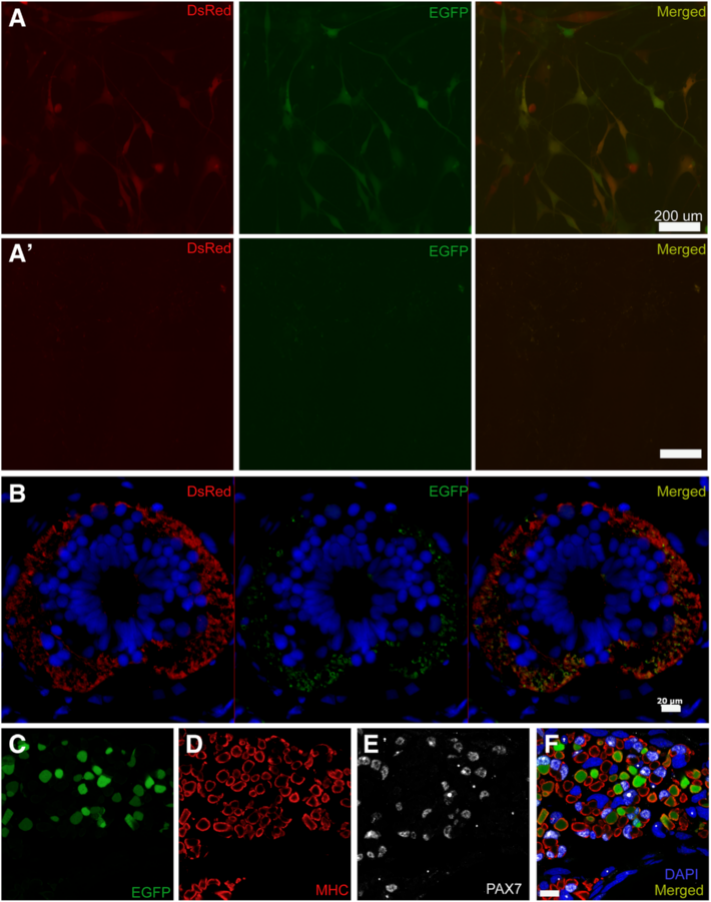
**Multiple genes transfer and tissue specific expression via foamy viruses. A**. EGFP and DsRED expression is seen in A1 cells transduced with mixture of EGFP and DsRED foamy viruses. A’. untransduced A1 cells. **B**. Cross section of tail showing spinal cord and surrounding nerve tracts. Tail blastema was transduced with mixture of FV containing DsRed and EGFP FV particles. Colocalization is seen between EGFP and DsRed fluorescent signal.**C**-**F**. Muscle-specific expression was achieved when FV vector particles harboring the Xenopus *CarAct:EGFP* cassette were injected into the blastema at day 5 after amputation of a 3 cm larvae. **C**. Cross section of limb showing EGFP expression in muscle tissue. **D**. MHC immunostaining of section to highlight muscle cells/fibers. **E**. PAX7 immunostaining of same section to delineate satellite cells. **F**. Overlay to show that GFP signal is exclusive of PAX7+ satellite cells. Scale bars: A,A’: 200 μm, B: 20 μm, C-F: 50 μm.

To ascertain the efficiency of FV to transfer two genes (via two separate foamy viruses), A1 cells were transduced with a mixture of FV (EGFP and DsRed). We saw colocalization of both the fluorescent proteins in majority of cells (Figure 5A). Similarly, colocalization of EGFP and DsRed was also observed in nerve tracts of spinal cord when a tail blastema was injected with the two FV viruses (Figure 5B). These results confirm that multiple FV particles can transfer genes in the same target cell.

To determine if viral transduction could be used to achieve cell type-specific expression, we inserted the *CarAct:EGFP* expression cassette (muscle specific promoter driving EGFP) into a FV vector and produced concentrated vector stocks as described in methods and materials. Subsequently, vector particles were injected in blastemas at day 5 and regeneration was allowed to occur. We observed EGFP expression only in the limb muscle fibers in the regenerated limb and not in any other cell type (Figure 5C-F). Therefore, foamy virus vector-mediated transgene integration results in faithful, cell-type restricted expression.

These results indicate that FV particles can infect blastema cells with stable and persistent expression during regeneration and after amputation. Furthermore, tissue specific expression is achieved when combined with tissue specific promoter. FV vectors have previously been shown to transduce cells from species very distantly related to their natural hosts [12,13,15]. Electroporation of plasmid DNA has been successfully established in salamanders but it cannot be used for experiments that require long-term gene expression as electroporated DNA is episomal and dilutes out during cell division. Similarly, establishing germline transgenic axolotls is a tedious process requiring 12-15 months (Khattak et al; Stem Cell Reports-in press). The results of this study show that FV vectors are an effective gene delivery tool for non-mammalian models, such as the salamander, resulting in long-term transgene expression. Interestingly, not only in vitro but also in vivo, their efficiency and the stability of transgene production was superior to the currently most favored retroviral vector system based on lentiviruses pseudotyped by the glycoprotein of the rhabdovirus VSV. Availability of foamy virus gene transfer system together with recently reported psuedotyped retrovirus [8] for regeneration research, such as the axolotl/salamander system, will greatly expand the ability to test gene function during regeneration.

## Conclusions

We here report on the successful application of foamy virus as a vector for transgene integration and sustained expression during regeneration. Upon infection of the blastema, EGFP expression was observed in multiple cell types, and expression lasted throughout regeneration and persisted through a second round of regeneration. Both limb and tail blastema were transduced with foamy virus expressing EGFP/DsRed and resulted in wide range of cells labeled after limb/tail regeneration was complete. We also demonstrated the compatibility of this system with cell-type specific expression by incorporating the Xenopus cardiac actin promoter in the foamy virus vector. Our observation of widespread and stable expression with the foamy virus contrasts with the difficulty to achieve persistent *in vivo* transgene expression with concentrated lentivirus vectors.

## Methods

### Cells and culture conditions

The human kidney cell line 293T [20] and the human fibrosarcoma cell line HT1080 [21] were cultivated in Dulbecco’s modified Eagle’s medium (DMEM) supplemented with 10% heat-inactivated fetal calf serum and antibiotics. The newt myogenic cell line A1 [17] was cultivated in 70% Eagles minimal essential media supplemented with 10% fetal bovine serum, insulin and penicillin/streptomycin.

### Expression constructs

For this study a replication-deficient expression-optimized 4-plasmid vector system based on prototype FV (PFV) recently developed by our laboratory was used [22] (Figure 1A). It comprises three packaging plasmids containing expression-optimized ORFs for the PFV capsid precursor protein Gag (pcoPG4) [15], the PFV enzymatic precursor protein Pol (pcoPP) as well as the PFV glycoprotein precursor Env (pcoPE) [22] and the transfer vector puc2MD9 Ubi WPRE [15]. The transfer vector contains the minimal essential cis-acting viral sequences [7] that are required for vector RNA and enzyme (Pol) encapsidation as well as vDNA synthesis and its integration into the host cell genome mediated by the *pol*-encoded viral reverse transcriptase and integrase, respectively. With FVs having one of the largest viral genomes (~12 kb) the PFV transfer vector can accommodate up to 9 kb of non-viral genetic material [23]. All packaging plasmids are based on the pczi expression vector, a pcDNA3.1+ variant containing an hCMV intron A sequence resulting in the expression of spliced mRNAs [24]. Expression-optimization and gene synthesis of the PFV ORFs was performed at Geneart and enables production of vector supernatants with 5-10 fold higher titers than achieved with FV vector systems based on authentic viral sequences [22,24,25]. The puc2MD9 Ubi WPRE transfer vector used in this study is a variant of puc2MD9 published previously having the original SFFV U3 promoter driving EGFP or DsRed marker gene expression replaced by a human ubiquitin C promoter (hUbiC) [15]. Furthermore the WPRE posttranscriptional active element was inserted downstream of the EGFP gene.

For generation of replication-deficient lentiviral vectors a 3-plasmid system was used (Figure 1B). It consists of two packaging plasmids, pCD/NL-BH [26], encoding HIV-1 Gag/Pol as well as some accessory proteins; pczVSV-G [27], encoding the vesicular stomatitis virus glycoprotein (VSV-G); and the HIV-1 transfer vector p6NST90 [15]. p6NST90 is a variant of p6NST50 [28] having the SFFV U3 driven IRES EGFP-Zeo expression cassette replaced by a hUbiC promoter driven EGFP/DsRed cassette. Details on the construction of the vectors are available on request.

### Generation of viral supernatants

FV supernatants containing recombinant viral particles were generated essentially as described previously [25,29]. Briefly, FV supernatants were produced by polyethyleneimine (PEI)-mediated cotransfection of 293T cells with transfer vector (e.g. puc2MD9 Ubi WPRE), Env- (pcoPE), Pol – (pcoPP), and Gag packaging plasmid (pcoPG4) at a ratio of 28:1:2:4 using 16 μg total DNA per 10-cm dish. At 32 h post transfection the medium was replaced, and cell-free viral supernatants were harvested by sterile filtration (0.45 μm pore size) an additional 16 h later. Supernatants were used directly or aliquots snap frozen on dry ice and stored at -80°C until further use. Lentiviral supernatants were generated by cotransfection of transfer vector (p6NST90), Gag/Pol packaging plasmid (pCD/NL-BH), and an Env packaging plasmid (pczVSV-G) at a ratio of 1:1:1 and harvested as described above. For in vivo transduction experiments vector particles were concentrated from cell-free viral supernatant by ultracentrifugation for 1.5 h at 25,500 rpm using a SW28 rotor (35 ml supernatant per bucket). Pelleted vector particles were gently resuspended on ice in PBS with 0.1 % BSA and aliquots were snap frozen and stored in – 80**°**C until use. Recovery of lentiviral VSV-G pseudotypes was generally above 70% and of PFV vector particles above 50%.

### Analysis of in vitro and in vivo transduction efficiency

Infectious titers used for calculation of multiplicities of infection (MOI) were determined on HT1080 cells. In vitro transductions of recombinant EGFP expressing FV or HIV-1 vector particles were performed by infection of 2 × 10^4^ HT1080 cells, plated 24 h in advance in 12-well plates or by infection of 2 × 10^4^ A1 cells, plated 48 h in advance in 6-well plates. For the infection 1 ml (HT1080) or 1.5 ml (A1) of the viral supernatant or dilutions thereof were incubated with the target cells for 8 h. The percentage of EGFP-positive cells was determined by fluorescence-activated cell sorter (FACS) analysis at different time points after infection, as indicated. For double infections, DsRed and EGFP viruses were mixed before putting them on A1 cells as indicated above. All transduction experiments were performed in triplicates and at least three times.

For in vivo infections, 2-3 cm axolotl larvae were used. The animals were bred and raised in our colony and kept in local tap water at 18°C. The larvae were fed with freshly hatched brine shrimps (Artemia) every day. All animal procedures were done according to the animal and biological safety level 2 (S2) license (24D-9168.11-9-2006-1 and 55-8811.72/34-16) issued by “*Regierungspräsidium Dresden”* and “*Sächsisches Staatsministerium für Umwelt und Landwirtschaft* (SMUL)” respectively. The ethical approval was obtained from the "Landesdirektion" for animal welfare for the State of Saxony. Before every animal procedure, animals were anesthetized in 0.01% ethyl-*p*-aminobenzoate (benzocaine; Sigma). For each experiment, lower forelimbs or tails of nine animals were amputated and animals were returned to fresh water in separate boxes for blastema induction. After five days, 5 day blastemas were injected with 0.5 – 2 μl of concentrated foamy and/or lentivirus with help of glass capillaries (Harvard Apparatus) pulled by using a Sutter Flaming Brown P-97 puller to a tip size of approximately 1–2 μm and injected using PV830 Pneumatic PicoPump (WPI). The transduced animals were kept on a wet tissue for 20 min before putting them in fresh water, which was changed every second day. EGFP/DsRed fluorescence was first observed 7-8 days post infection. Amputation of limbs and detection of fluorescence was performed on Olympus SZX16 stereomicroscope. All transduction experiments were repeated three times.

### Immunohistochemical analysis of transduced re-regenerated limb tissue

Regenerated limbs were amputated 28 days post infection to harvest the limb tissue. The cut part was fixed in 1% MEMFA for 4 hrs at RT followed by three washes of PBS and then incubated overnight at 4°C in 30% sucrose. The next day, tissue was embedded in Tissue-Tek (OCT compound-Sakura), cryosectioned and slides were allowed to dry at RT for 1-2 hrs. Limb and tail sections were washed 3 times with PBS-Tween (0.3%) and blocked for one hour with 2% BSA, 5% normal serum and 0.3% Triton-X (blocking solution), followed by overnight incubation with the primary antibody diluted in blocking solution. Primary antibodies used were anti-PAX7 mAB (Developmental Studies Hybridoma Bank), anti-MHC (monoclonal antibody 4A1025 kindly provided by S. Hughes), mouse anti-chicken collagen Type II antibody (Millipore) and monoclonal anti-Pan Cytokeratin antibody mixture (Sigma). After several washes with PBS-Tween, sections were then incubated for 1 hour with respective secondary antibodies and Hoechst solution for nuclear staining. Images were taken on Leica confocal microscope and Zeiss Observer.

## Competing interests

The authors declare that they have no competing interests.

## Authors’ contributions

SK, TSG, NS, SP performed experiments; SK, EMT and DL designed the experiments, analyzed data and wrote the manuscript. All authors read and approved the final manuscript.
